# Pneumonia burden in elderly patients: a classification algorithm using administrative data

**DOI:** 10.1186/1471-2334-13-559

**Published:** 2013-11-25

**Authors:** Silvia Cascini, Nera Agabiti, Raffaele Antonelli Incalzi, Luigi Pinnarelli, Flavia Mayer, Massimo Arcà, Danilo Fusco, Marina Davoli

**Affiliations:** 1 Department of Epidemiology, Regional Health Service, Via di S.Costanza 53, 00198, Lazio Region, Rome, Italy; 2 Department of Geriatric Medicine, Campus University, Rome, Italy

**Keywords:** Pneumonia, Elderly, Healthcare database

## Abstract

**Background:**

Pneumonia has traditionally been classified into two subtypes: community-acquired pneumonia (CAP) and nosocomial pneumonia (NP). Recently, a new entity has been defined, called healthcare-associated pneumonia (HCAP). Few studies have investigated the potential of population-based, electronic, healthcare databases to identify the incidences of these three subtypes of pneumonia. The aim of this study was to estimate the burden of the three subtypes of pneumonia in elderly patients (aged 65+ years) in a large region of central Italy.

**Methods:**

A retrospective cohort study was performed using linked regional Hospital Information System and Mortality Register. All episodes of pneumonia in elderly patients, who were discharged from the hospital in 2006–2008, were selected for the study. Following a validated ICD-9-coding algorithm, incidents of pneumonia events were classified into three groups (HCAP; probable nosocomial pneumonia, PNP; and CAP). Hospitalisation rates were calculated by age group (65–79, 80+), gender, and year, using the population from the Institute of Statistics (ISTAT) census estimates as denominators.

**Results:**

A total of 26,239 pneumonia events occurred in 24,338 patients residing in the Lazio region, aged 65+ years: 2257 HCAP, 6775 PNP, and 17,107 CAP. For all subtypes, the proportion of males was greater than females. Comorbidity status was more severe in HCAP than in the other categories. In-hospital mortality, 30-day mortality, and length of hospital stay were twice higher in HCAP than in CAP episodes. The annual incidence rates were 0.7, 2.1, and 5.4 episodes per 1000 residents for HCAP, PNP, and CAP, respectively. From 2006 to 2008, incidence rates slightly increased for all three subtypes.

**Conclusion:**

Health care databases can be used to give a timely and inexpensive picture of the epidemiology of pneumonia. HCAP represents a distinct category of pneumonia, with the longest stay, highest mortality, and the greatest comorbidity.

## Background

Acute lower respiratory infections are a leading cause of death worldwide and a primary source of morbidity and mortality in older adults [1]. Among them, pneumonia is variably classified as either community acquired (CAP) or nosocomial pneumonia (NP), depending upon the time and setting of onset. Nosocomial pneumonia refers to infections acquired during hospitalisation, and has been differentiated into ventilator-associated pneumonia or hospital-acquired pneumonia, depending on whether the infection developed during the course of mechanical ventilation or was not associated with ventilation, respectively.

Healthcare reflects a continuum of care, with many traditional inpatient services provided in outpatient settings. Many invasive therapies are administered in nursing homes and rehabilitation hospitals, and minor surgery is frequently performed in outpatient centres. Moreover, some patients reside more often in health care structures than in the community. As a result, the traditional distinction between community and nosocomial infection has become less clear in recent years. For this reason, in 2005, the American Thoracic Society (ATS)/Infectious Diseases Society of America (IDSA) defined a new category of pneumonia: healthcare-associated pneumonia (HCAP) [2]. This new category includes patients with pneumonia who have had recent contact with the healthcare system.

The incidence and prognosis of HCAP has increasingly been recognised and described in various contests [3-7]. In many studies, however, the criteria for the classification of the three forms of pneumonia are not consistent with the ATS definition [4-8]. Differences in the case identification strategy across studies make it difficult to compare incidence estimates [2,4-8]. Electronic health datasets, like mortality or hospitalisation registers, are increasingly used for epidemiological purpose, and, in particular, to estimate the burden of diseases [9-13]. They are readily available and easy to access, despite problems of completeness and accuracy [14,15].

Few studies have investigated the potential of population-based, electronic healthcare databases to identify pneumonia [4,5,7,8]. Specifically, their usage and experience in the European continent is limited [7,8,16,17]. The aim of this study was to estimate the burden of the three different subtypes of pneumonia (HCAP, NP, and CAP) in elderly people (65+ years of age), using healthcare databases in the Lazio region of central Italy.

## Methods

### Design and data sources

Different health information systems were used to study the population (over 5,700,000 residents) of the Lazio region. The Hospital Information System (HIS) of the Lazio region routinely collects data from all Lazio hospitals, including patient demographic data, admission referral source, discharge status, up to six discharge diagnoses (ICD-9-CM), up to six hospital procedures (ICD-9-CM), and the regional code of the facility. The inter-regional mobility dataset collects data on all the hospitalisations of Lazio residents that occur outside the region. The Outpatient Specialist Service Information System (OSSIS) collects data from outpatient clinics (visits, specialist services instrumental and laboratory diagnostic results). The drug-dispensing registry (PHARM) comprises individual records for each medical prescription that is dispensed from public and private pharmacies. The registry is limited to drugs dispensed to outpatients, who are reimbursed by the healthcare system. Drugs are identified by the national drug register code. Individual patient data and date of dispensing are reported for each prescription. The Regional Registry of Causes of Death (ReNCaM) lists the causes of death, coded according to the International Classification of Diseases, 9th Revision, for all resident deaths in the region. For this study we also obtained information from the Regional Registry of Vaccination.

### Population

All episodes of patients who were treated for pneumonia, as the main or secondary diagnosis (ICD-9-CM codes are reported in Additional file 1), discharged from the hospital between January 1^st^ 2006 and December 31^th^ 2008, and who were residents of the Lazio region, were identified from the regional Hospital Information System (HIS) and inter-regional mobility dataset. Only the first of multiple admissions over 30 days for the same patient was included. Episodes of pneumonia, which referred to patients younger than 65 years of age, were excluded.

### Pneumonia subtype classification

Pneumonia events were classified into three subtypes through automated record linkage procedures between HIS and OSSIS, following the definitions by Venditti [6] and Giorgi Rossi [17]:

● HCAP: pneumonia in a patient who met at least one of these criteria: 1) patients who had dialysis or chemotherapy in the last 30 days before the indexed episode of care, 2) patients living in a nursing home or long-term care facility, 3) patients who had an admission to an acute-care hospital for at least two days or had surgery within the past 180 days [6]. Information on residence in a nursing home or long-term facility was not available for the years under study; therefore, only criteria 1 and 3 were applied.

● PNP (probable nosocomial pneumonia): principal diagnosis other than pneumonia, complications of pneumonia, or chronic obstructive pulmonary disease (COPD); diagnosis of trauma, with a secondary diagnosis of pneumonia [17].

● CAP: any other incident episode not included in the definition of PNP or HCAP.

### Demographic and clinical characteristics

Demographic characteristics and comorbidities (ICD-9-CM codes in any secondary diagnosis field) in the index admission or in two previous years hospitalizations were retrieved from HIS according to validated algorithms [18]. ICD-9-CM codes are reported in Additional file 1.

An area-based socio-economic index, calculated from the 2001 census data (including level of education, occupation, dwelling ownership, family size, and people/room density) was only available for residents of Rome. Five levels of the socio-economic index were defined; level 1 refers to the population at the highest socio-economic position, while level 5 refers to the population at the lowest socio-economic position [19].

Information on COPD and respiratory failure diagnoses within the previous two years was also retrieved from HIS, while the regional register provided information on influenza vaccinations received in the previous two years. Domiciliary oxygen usage within the previous year was obtained from PHARM [20].

Information on acute stroke was retrieved from the HIS in the same admission. Comorbid conditions were classified into the following categories: heart failure, diabetes, chronic kidney, malignant tumours, hypertension, ischemic heart, other heart disease, arrhythmias, cerebrovascular disorders, digestive, anemias/coagulopathies, neurological, psychiatric, chronic respiratory.

The severity of illness was ascertained through information on the use of continuous invasive mechanical ventilation (any procedures) or admission, discharge, or transition to the intensive care unit in the same admission. The type of hospital was classified into three levels: public, teaching, or private.

We recorded information of in-hospital mortality from HIS, while 30-day mortality (death within 30 days of admission date) information was obtained through a record-linkage procedure with the ReNCaM; the date of admission was considered for the start of follow-up. For each record, length of stay was calculated by the difference between the date of discharge (or death) and date of admission.

### Statistical analysis

Hospitalisation rates for the three pneumonia subtypes were calculated by age groups (65–79, 80+) and gender, using the total number of residents on January 1st 2008 as the denominator, taken from the Institute of Statistics (ISTAT, 2008) census estimates (1,056,000 inhabitants over 65 years of age). Rates were expressed as the average annual incidence of hospitalisations per 1000 residents. Moreover, hospitalisation rates for the three subtypes were calculated for each year (2006, 2007, 2008), according to age groups (65–69, 70–74, 75–79, 80–84, 85–89, 90+), dividing the number of new cases per year by the number of persons-at-risk per year, per 1000 residents. The level of significance was set at 5% (*p* < 0.05), and all analyses were performed using SAS Version 9.2.

According to the Regional Law, the present study, which was based on anonymous computer records from health information systems, did not require ethical approval.

## Results

A total of 26,239 pneumonia events occurred in 24,338 patients, aged 65+ years: 2257 (8.6%) HCAP, 6775 (25.8%) PNP, and 17,107 (65.2%) CAP. Among those with HCAP, 1172 (51.9%) had pneumonia within 10 days of residing at the hospital. Table 1 shows the characteristics of the study population. Males were more prevalent than females across all three subtypes of pneumonia. Socio-economic status did not distinguish subtypes. People with HCAP or CAP were more likely to use long-term oxygen therapy in the previous year, and to have a diagnosis of COPD and respiratory failure than people with PNP. For acute strok” in the same admission, there were evident differences between pneumonia subtypes (HCAP, 4.3%; PNP, 8.5%; and CAP, 0.8%). At least one comorbidity was present in 69% of all episodes. All comorbidities, except for heart failure, arrhythmias, and digestive diseases, were more prevalent in HCAP than in CAP or PNP patients.

**Table 1:** Demographic and clinical characteristics, according to pneumonia subtype

	**HCAP**		**PNP**		**CAP**		**Chi-square test**
	**n = 2257**		**n = 6775**		**n = 17107**		
	**n** ***%***		**n** ***%***		**n** ***%***		***p value***
***Gender***							
Females	939*(41.6)*		3236*(47.8)*		7704*(45.0)*		*<0.001*
Males	1318*(58.4)*		3539*(52.2)*		9403*(55.0)*		*<0.001*
***Residence***							
Rome	1422*(63.0)*		4231*(62.5)*		10143*(59.3)*		*<0.001*
Province of Rome	452*(20.0)*		1394*(20.6)*		3715*(21.7)*		*<0.001*
Rest of Lazio	383*(17.0)*		1150*(17.0)*		3249*(19.0)*		*<0.001*
***Age***							
65-69	384*(17.0)*		724*(10.7)*		2152*(12.6)*		*<0.001*
70-74	486*(21.5)*		1052*(15.5)*		2894*(16.9)*		*<0.001*
75-79	561*(24.8)*		1428*(21.1)*		3791*(22.2)*		*<0.001*
80-84	398*(17.6)*		1591*(23.5)*		3810*(22.3)*		*<0.001*
85-89	287*(12.7)*		1170*(17.3)*		2629*(15.4)*		*<0.001*
90+	141*(6.25)*		810*(11.9)*		1831*(10.7)*		*<0.001*
***Socioeconomic position (Rome residents only)***							
1 (high)	210*(9.3)*		687*(10.1)*		1483*(8.7)*		*<0.001*
2	247*(10.9)*		640*(9.4)*		1591*(9.3)*		*<0.001*
3	231*(10.2)*		702*(10.4)*		1695*(9.9)*		*<0.001*
4	239*(10.6)*		684*(10.1)*		1664*(9.7)*		*<0.001*
5 (low)	238*(10.5)*		655*(9.7)*		1815*(10.6)*		*<0.001*
***Respiratory conditions***							
Oxygen therapy (previous year)	279*(12.3)*		756*(11.2)*		2219*(13.0)*		*0.001*
COPD (previous two years)	494*(21.9)*		1122*(16.6)*		3555(20.8)		*<0.001*
Respiratory failure (previous two years)	242(10.7)		453(6.7)		1620(9.5)		*<0.001*
Influenza Vaccination (previous two years)	1218*(54.0)*		3820*(56.4)*		6955*(40.7)*		*<0.001*
***Acute stroke (same admission)***	97*(4.3)*		573*(8.5)*		145*(0.8)*		*<0.001*
***Comorbidities (previous two years)***							
Heart failure	551*(24.4)*		2073*(30.6)*		3461*(20.2)*		*<0.001*
Diabetes	415*(18.4)*		902*(13.3)*		2217*(13.0)*		*<0.001*
Chronic kidney disease	632*(28.0)*		1121*(16.5)*		2313*(13.5)*		*<0.001*
Malignant tumours	721*(31.9)*		1397*(20.6)*		2148*(12.6)*		*<0.001*
Hypertension	827*(36.6)*		1698*(25.1)*		4327*(25.3)*		*<0.001*
Ischemic heart disease	583*(25.8)*		1573*(23.2)*		3882*(22.7)*		*0.004*
Other heart disease	528*(23.4)*		1287*(19.0)*		2835*(16.6)*		*<0.001*
Arrhythmias	602*(26.7)*		1986*(29.3)*		4267*(24.9)*		*<0.001*
Cerebrovascular disorders	745*(33.0)*		2023*(29.9)*		4251*(24.8)*		*<0.001*
Digestive disease	139*(6.2)*		540*(8.0)*		997*(5.8)*		*<0.001*
Anaemias/coagulopathies	403*(17.9)*		883*(13.0)*		1972*(11.5)*		*<0.001*
Neurological diseases	356*(15.8)*		914*(13.5)*		1951*(11.4)*		*<0.001*
Psychiatric diseases	217*(9.6)*		631*(9.3)*		1714*(10.0)*		*0.243*
Chronic respiratory diseases (other than COPD)	113*(5.0)*		378*(5.6)*		970*(5.7)*		*0.435*
***Severity of disease (same admission)***							
Ventilation	417*(18.5)*		207*(3.1)*		475*(2.8)*		*<0.001*
Intensive care	580*(25.7)*		367*(5.4)*		563*(2.8)*		*<0.001*
***Type of hospital***							
Public	1361*(60.3)*		4485*(66.2)*		11297*(66.0)*		*<0.001*
Teaching	598*(26.5)*		1391*(20.5)*		2967*(17.3)*		*<0.001*
Private	298*(13.2)*		899*(13.3)*		2843*(16.6)*		*<0.001*
***Mortality***							
In hospital	729*(32.3)*		1667*(24.6)*		2269*(13.3)*		
30 day-mortality	863*(38.2)*		1916*(28.3)*		2710*(15.8)*		
	*Mean*	*Median*	*Mean*	*Median*	*Mean*	*Median*	
***Length of hospital stay***	27.1	*17.0*	16.8	*13.0*	13.2	*10.0*	

The hospital-type distribution of where patients were treated varies across the three subtypes. In-hospital and 30-day mortality for HCAP were twice that of CAP; the corresponding figures for PNP were intermediate between those of HCAP and CAP, but closer to HCAP. A similar pattern was observed for length of stay; the length of stay for HCAP episodes was twice that of CAP, with intermediate lengths for PNP episodes.

The most common aetiological agents, based on ICD-9-CM codes, are reported in Table 2. For over 76% of all episodes of pneumonia, the specific organism was unknown. Among the remaining 24% of cases, some differences in the aetiology were evident; HCAP was more frequently caused by *Pseudomonas* and *Staphylococcus aureus*.

**Table 2:** Pneumonia aetiology, based on ICD-9_CM codes

**Aetiology**	**HCAP**	**PNP**	**CAP**
	**n = 2257**	**n = 6775**	**n = 17107**
	**n%**	**n%**	**n%**
Bronchopneumonia, organism unspecified	903(40.0)	3482(51.4)	8148(47.6)
Pneumonia, organism unspecified	637(28.2)	1830(27.0)	4901(28.6)
Pneumonia, specified organisms			
*Bacterial pneumonia, unspecified*	*330*(14.6)	*863*(12.7)	*2299*(13.4)
*Pneumonia due to Pseudomonas*	*82*(3.6)	*44*(0.6)	*200*(1.2)
*Pneumonia due to other specified bacteria*	*59*(2.6)	*105*(1.6)	*247*(1.4)
*Pneumococcal pneumonia [Streptococcus pneumoniae]*	*45*(2.0)	*108*(1.6)	*289*(1.7)
*Methicillin-susceptible pneumonia due to Staphylococcus aureus*	*32*(1.4)	*16*(0.2)	*99*(0.6)
*Pneumonia due to Klebsiella pneumoniae*	*17*(0.7)	*26*(0.4)	*93*(0.5)
*Pneumonia due to other gram-negative bacteria*	*15*(0.7)	*10*(0.1)	*80*(0.5)
*Pneumonia due to Staphylococcus, unspecified*	*15*(0.7)	*19*(0.3)	*122*(0.7)
*Pneumonia due to Escherichia coli [E. coli]*	*13*(0.6)	*9*(0.1)	*54*(0.3)
*Respiratory conditions due to chemical fumes and vapours*	*14*(0.6)	*89*(1.3)	*23*(0.1)
*Other*	*95*(4.3)	*174*(2.6)	*552*(3.2)

The average annual incidence rate (given in number of episodes per 1000 inhabitants) for HCAP was 0.7; for PNP, 2.1; and for CAP, 5.4 (3.7 for those aged 65–79 years, and 10.4 for those aged 80+ years) (Table 3). The incidence increased with age.

**Table 3:** Average Annual Incidence rates of pneumonia hospitalisations per 1000 persons, Lazio, 2006-2008

**Gender**	**HCAP**									**PNP**									**CAP**								
	**65-79**	**95% CI**		**80+**	**95% CI**		**Total**	**95% CI**		**65-79**	**95% CI**		**80+**	**95% CI**		**Total**	**95% CI**		**65-79**	**95% CI**		**80+**	**95% CI**		**Total**	**95% CI**	
Males	0.9	0.8	0.9	1.5	0.5	1.7	1.0	0.9	1.1	1.8	1.7	1.9	5.9	5.6	6.2	2.7	2.6	2.8	5.1	5.0	5.2	14.9	14.4	15.3	7.1	7.0	7.2
Females	0.4	0.4	0.4	0.8	0.2	0.9	0.5	0.5	0.5	1.0	0.9	1.0	3.7	3.6	3.9	1.8	1.7	1.8	2.6	2.6	2.7	8.1	7.8	8.3	4.2	4.1	4.3
**Total patients**	0.6	0.6	0.6	1.0	0.3	1.1	0.7	0.7	0.7	1.4	1.3	1.4	4.5	4.3	4.6	2.1	2.1	2.2	3.7	3.6	3.8	10.4	10.2	10.6	5.4	5.3	5.5

**Figure 1:**
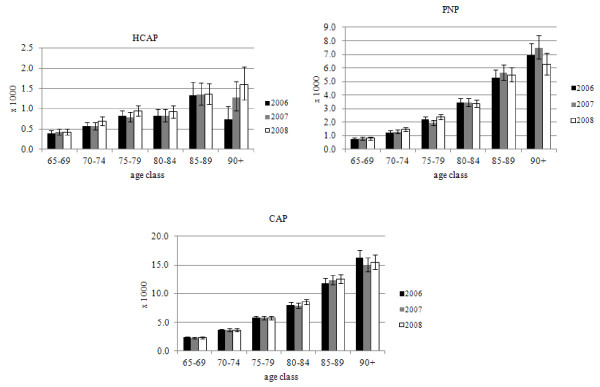
Incidence of pneumonia subtypes per 1000 persons per year, according to age group.

The distribution of the incidences of the three subtypes of pneumonia during the study period is presented in Figure 1. The incidences of all three subtypes of pneumonia slightly increased over the three years of the study. For HCAP, the incidence per 1000 residents increased from 0.6 in 2006 to 0.8 in 2008; for PNP, from 2.1 in 2006 to 2.2 in 2008; and from 5.3 in 2006 to 5.5 in 2008 for CAP. A statistically significant increase in the incidence rate was found among the very old (90+ years of age) with HCAP (2006: 0.7, 95% CI: 0.5-1.1; 2008: 1.6, 95% CI: 1.2-2.0 per 1000 inhabitants), an effect that was not evident in the other two subtypes. However, in 2006 and 2007, a low proportion of people over 90+ years of age had episodes of HCAP.

## Discussion

Results from the present study suggest that the three types of pneumonia are characterized by important differences in demographic/social and clinical profiles, as well as in their impact on healthcare. This finding is of special interest, as such an algorithm can be easily implemented in most Western countries, thus, allowing for the analysis of the impact of pneumonia on the healthcare system, as well as the effect of public health measures. Indeed, actions preventing or limiting HCAP or PNP are on going in many health care systems [21].

We found that HCAP has a dramatic economic and health effect, as reflected by the longest stay and the highest mortality rate in our study. The most severe comorbidity status of this group likely accounted, at least to some extent, for such an unfavourable outcome. However, biological characteristics, such as the microbial patterns, might also be a cause. Indeed, HCAP has been reported to have a complex polymicrobial origin in most instances [5]. Unfortunately, our database could only test this hypothesis to a limited extent. Claims data have a limited potential in their ability to identify pneumonia aetiology. Indeed, pathogenic agents should be detected in respiratory samples (i.e., sputum, bronchoscopically-retrieved samples), and correctly coded. On the other hand, using the distribution of the variable “acute stroke” as a proxy of aspiration pneumonia, we could detect important differences between pneumonia subtypes.

The HCAP pneumonia category is defined in order to distinguish pneumonia that occurs in patients with frequent contact with the healthcare system, in terms of hospitalisation, hemodialysis, or residency in a long-term residence facility. However, the definition of HCAP and the relative frequencies of HCAP-defining subgroups are highly variable, depending on the healthcare system, distribution of facilities, provision of care, insurance systems, and population distribution [22]. This variability makes it difficult to compare occurrence estimates across different countries.

In Italy, healthcare is provided to all citizens and residents by a mixed public-private system. The public part is the National Health Service (NHS), which is organized under the Ministry of Health, is administered on a regional basis, and provides universal health insurance for residents. However, in-home care services are not widely and homogenously diffuse in Italy. In Lazio, about 6000 residents, who are over 65, reside in nursing homes (about 5.7 per 1000 inhabitants). In 2010, the acute hospitalisation rate, standardized for age and gender, was around 115 per 1000 inhabitants, and respiratory diseases are among the most prevalent causes of hospitalisation in the elderly [23]. On the other hand, in Japan, there are many elderly people who receive in-home care services [24]. In the United States, there is a mixed system, containing elements of both traditional sickness insurance and national health coverage [5].

These important differences in healthcare availability among countries likely account for differences in disease categories, such as HCAP, which largely depend upon the healthcare organisation. Accordingly, our data about HCAP are worthy of comparison with those obtained in other countries. Analogous to previous epidemiological studies, incidence of CAP was strongly related to age, and peaked in the very old, whereas HCAP had the highest mortality, as HCAP patients are, by definition, more debilitated by comorbidities [4,7,21]. This finding seems interesting because the geriatric nature of our population theoretically had the potential for smoothing the relationship between age and incidence of CAP.

Strengths of this study are the population-based design, the large sample size, and the use of already existing, readily available data sources. Furthermore, the methodology used in this study, and the algorithm used to define incident cases of pneumonia, were derived from previous epidemiological studies [6,7,17]. The estimates for CAP incidence in this study are slightly higher than those found in a previous study, which was conducted in 1997–98 in the same geographical area, using similar methodology (for those aged 65+ years, the incidence rate was 480/100,000 residents) [17]. In a German study, which was conducted on behalf of a performance measurement program of healthcare quality, with data from over 380,000 individuals in 2005–2006, the mean incidence in people aged 60+ years was 7.7 per 1000 inhabitants over two years [16].

Our study has several limitations. First, we could not determine the presence or absence of the pneumonia at the time of admission to the hospital. For this reason, we modified the definition of NP to “probable” NP (PNP). Furthermore, in the Lazio region, during the years of the study, information on in-home care or residency in long-term facilities was not available, and this may have resulted in the underestimation of HCAP cases. To quantify this critical point, we performed a record-linkage analysis between the hospital information system and the home residence database for the year 2011. In fact, the information on in-home care residency is only available in the Lazio region for 2011. We found only 150 hospitalisations for pneumonia in nursing home dwellers in the 180 days before admission. From these data, we are quite confident that the lack of data on residency in nursing or long-term care facilities has had a minimal impact on the subtype classifications in our study. Finally, the quality of data is a crucial limitation of this study, due to the known potential inaccuracy of administrative data [25,26]. The algorithm is very sensitive to the quality of ICD-9-CM coding. Despite the lack of a validation study, we consider the performance of the diagnostic codes used in our study to be good. In a previous “re-abstract study” of a random sample of over 390 clinical charts in our region, a good level of accuracy in pneumonia registration was observed (confirmation rate 80%; high level of specificity) [27]. Recently, in a study conducted in the USA, the ICD-9-CM codes for pneumonia demonstrated their validity as markers of this condition (positive predictive value was 88%).

## Conclusion

In conclusion, administrative databases can be used to give a timely and comprehensive picture of the epidemiology of pneumonia. They can also be used for epidemiological and health policy purposes. It is desirable that as many countries as possible, at least with homogeneous economic and societal statuses, share both administrative databases and classifying algorithms, in order to compare the epidemiology of pneumonia, and the effects of common or different healthcare initiatives. As the population of older adults and the prevalence of medical conditions that predispose to pneumonia increase, hospitalisations for pneumonia are likely to continue to increase, unless effective interventions are implemented. Results from large epidemiological studies, such as this study, based on inexpensive databases, might help to implement and monitor public health actions.

## Competing interests

The authors declare that they have no competing interests.

## Authors’ contribution

SC contributed to the conception and design of the study, interpreted the results, analysed the data drafted the article. NA, RAI, contributed to the conception and design of the study, interpreted the results and drafted the article. LP, MD contributed to the conception and design of the study and interpreted the results. FM, MA and DF analysed the data and interpreted the results. All authors read and approved the final manuscript.

## Pre-publication history

The pre-publication history for this paper can be accessed here:

http://www.biomedcentral.com/1471-2334/13/559/prepub

## Supplementary Material

Additional file 1 ICD-9-CM codes used to identify episodes of pneumonia and to define comorbidities. Click here for file
