# Supplementary material for: Pneumonia burden in elderly patients: a classification algorithm using administrative data
Source: BMC Infect Dis. 2013 Nov 25;13:559. doi: 10.1186/1471-2334-13-559 (PMC4222665; doi:10.1186/1471-2334-13-559)
Supplement: Additional file 1 — ICD-9-CM codes used to identify episodes of pneumonia and to define comorbidities. [file 1471-2334-13-559-S1.doc]

ICD-9-CM codes used to identify episodes of pneumonia and to define comorbidities:

The following ICD-9-CM codes were used to identify episodes of patients treated for pneumonia, as the main or secondary diagnosis:

| Pneumonia |  | 480-486 |  |  |
| --- | --- | --- | --- | --- |
| Pneumonia due to solids |  | 507 |  |  |
| and liquids |  |  |  |  |
| Pulmonary tularemia |  | 021.2 |  |  |
| Pulmonary actinomycosis |  | 039.1 |  |  |
| Varicella pneumonia |  | 052.1 |  |  |
| Postmeasles pneumonia |  | 055.1 |  |  |
| Ornithosis with pneumonia |  | 073.0 |  |  |
| Candidias of lung |  | 112.4 |  |  |
| Primary coccidiomycosis |  | 114 |  |  |
| Toxoplasmosis |  | 130.4 |  |  |
| Pneumocystis carinii |  | 136.3 |  |  |
| Influenza with pneumonia |  | 487.0 |  |  |
| Salmonella Pneumonia |  | 003.22 |  |  |
| Histoplasmosis with pneumonia |  | 115.05, 115.15, 115.95 | | |

The validated algorithm to define comorbidities in the index admission or in two previous years comprised the following conditions:
